# Polydeoxyribonucleotide Dermal Infiltration in Male Genital Lichen Sclerosus: Adjuvant Effects during Topical Therapy

**DOI:** 10.1155/2013/654079

**Published:** 2013-12-30

**Authors:** Luigi Laino, Silvia Suetti, Isabella Sperduti

**Affiliations:** ^1^Dermatology and Venereology Center, Via Bixio 95, 00185 Rome, Italy; ^2^Regina Elena National Cancer Institute, Via Elio Chianesi 53, 00144 Rome, Italy

## Abstract

*Background*. Lichen sclerosus (LS) is an autoimmune inflammatory skin disease that leads to tissue sclerosis. Actually, the first-line treatment consists of local steroid as clobetasol propionate (CP). Polydeoxyribonucleotide (PDRN) has demonstrated anti-inflammatory effects through the reduction of cytokine production and growth stimulation of fibroblast. *Objective*. To evaluate the efficacy of intradermal administration of PDRN in male patients suffering from genital lichen sclerosus in addition to topical 0.05% CP, as compared to administering 0.05% CP without PDRN injection. *Patients/Methods*. A group of male patients (*n* = 28; aged 25 to 65) suffering from LS were observed during topical therapy or subdermal in addition to topical therapy. Disease activity at baseline was evaluated on Investigator's Global Assessment (IGA) and the Dermatology Life Quality Index (DLQI). We used polydeoxyribonucleotide in a commercial preparation for human use and a topical CP emulsion. *Results*. After therapy, in all group A patients there has been a regression of most of clinical pathological signs, while there has been a moderate improvement in all group B patients. *Conclusions*. On site intradermal administration of PDRN, associated with CP 0.05% cream, seemed to be associated with a clinical improvement of lichen sclerosus better than CP used in single therapy.

## 1. Introduction

Several researchers have shed new light on the importance of the action of extracellular nucleotides and nucleosides in increasing cell proliferation and reducing inflammation. PDRN, an A2A adenosine receptor, acts as mitogen for fibroblasts, endothelial cells, and preadipocytes [1, 2] working with different growth factors (VEGF, PGF, and FGF). PDRN is used in plastic and dermatologic surgery, and recently in urology, for its regenerative properties, restorative effects in ischemic skin flaps [3], and being used to improve intratesticular vascularisation [4]. Recently, the effects of PDRN have been analysed in a number of tissues, such as corneal epithelium [5], human bone [6], and skin [3]. PDRN is involved in protective and regenerative effects on UV-irradiated mouse cell cultures [7] and UV-irradiated dermal fibroblast [8]. PDRN has shown proliferation effects in human preadipocytes, which represent the richest reservoir of human adult stem cells [9]. In the light of these preliminary results, and because of these specific properties, we decided to perform the clinical observation, before and after therapy, of a local subdermal administration of PDRN in lichen sclerosus genital lesions, focusing on the anti-inflammatory and positive regenerative effects of this A2A adenosine receptor. CP, a potent corticosteroid with marked anti-inflammatory action, has emerged as a topical medication for the treatment of inflammatory and immunological skin disorders, such as acute dermatitis and chronic dermatosis, such as lichen ruber planus, psoriatic plaques, and lichen sclerosus. After 4 months of therapy, according to IGA and DLQI assessment, all patients (group A; *n* = 14) treated with PDRN + CP showed drastic improvement of the various signs of the disease, related to different aspects of LS, such as hypertrophy, atrophy, leukoplakia, erosion, pigmentation, and inflammation, as compared to patients (group B; *n* = 14) treated with topical CP therapy.

## 2. Materials and Methods

### 2.1. Materials

We used polydeoxyribonucleotide in a commercial preparation for human use, containing 5625 mg of PDRN in a 3 mL ampoule sterilized at 121°C for 20 min. PDRN is a pure substance at 95%, constituted of different lengths of PDRN (from 50 to 2000 base pairs) obtained from sperm of salmon trout (*Oncorhynchus mykiss*) for human alimentation, through an original purification and sterilization process. In addition, we used a topical CP oil/water (O/W) emulsion.

### 2.2. Methods

We selected a group of male patients (*n* = 28) aged from 25 to 65, referred to our center with a history of Lichen sclerosus of genitalia, involving both prepuce and penis gland and with a moderate to severe impairment of sexual relationship. The main observed features of LS were erythema, infiltration, mucosal excoriation, lichenification, hypopigmentation, hyperpigmentation, and ecchymoses. We made a diagnosis of LS, both on the basis of clinical data and through biopsy, in compliance with the recent guidelines of the British Association of Dermatologists [10]. The patients were evaluated by the investigator on the Investigator's Global Assessment (IGA) (≥2 on a 4-point Likert scale; 0 = no disease—no inflammatory signs; 1 = mild disease—mild erythema, infiltration, lichenification, and excoriation; 2 = moderate disease—moderate erythema, infiltration, lichenification, and excoriation; 3 = severe disease—severe erythema, infiltration, lichenification, and excoriation) and the Dermatology Life Quality Index (DLQI) [11]. Exclusion criteria were phimosis, skin conditions at baseline that would interfere with LS evaluation, immunosuppression, malignancies, bacterial, viral, or fungal infection within 2 weeks prior to study initiation, and any prior intervention therapy (cryotherapy, laser, curettage, electrocautery, excision, or immunomodulators) in the last 2 months. We divided our patients into 2 groups, A (*n* = 14) and B (*n* = 14), with homogeneous IGA and DLQI. For group A patients, we proposed a therapy of 8 session. In every session we performed subdermal infiltrations with PDRN, corresponding to the lesions of lichen sclerosus and some neighboring areas. This was performed using a 30-gauge needle with application of anesthetic cream (lidocaine/prilocaine 25 mg + 25 mg cream) in occlusion for 15–20 minutes, in order to minimize discomfort during the intervention. In association, we prescribe a daily application of CP cream overnight. We performed 4 clinical controls (baseline, and monthly) with photo archive, before and after the infiltration sessions. As for group B patients (*n* = 14), we proposed the same topical therapy without any PDRN subdermal injections. All patients gave their informed consent to the treatment after an exhaustive explanation of effects, side or unwanted effects, especially related to ultrapotent corticosteroid topical application and subdermal administration of PDRN; all patients conduct rules before, during, and after treatment; in particular, the patients are informed that the clobetasol propionate—also in accordance with the latest guidelines published by British Association of Dermatologists [10]—is actually the only first-line therapy in course of lichen sclerosus and that polydeoxyribonucleotide solution—proposed by us only as adjuvant therapy—is currently available in Italy as drug suitable for subcutaneous infiltration; PDRN are recognized by Pharmacopoeia as “drugs without side or unwanted known effects” and no side or un wanted effects are actually described in the international scientific literature”.

We performed monthly clinical and photographic controls until 6 months after the end of the therapy; serologic parameters evaluations before starting therapy, during (every 1 month), and after therapy were performed by all patients.

### 2.3. Statistical Methods

Descriptive statistics were used to summarize pertinent study information. The associations were analyzed through the Fisher exact test, when appropriate. Comparisons between groups were carried out for different variables, using either Student's *t*-test or Mann-Whitney test. A Wilcoxon signed-rank test or Student's paired *t*-test was performed to evaluate the variations before and after therapy in each group. The level of significance was set at *P* = 0.05. SPSS software (version 20.0, Chicago, IL, USA) was used for all statistical analyses. We divided the patients into two groups (group A, *n* = 14; group B, *n* = 14) according to different therapy protocols.

## 3. Results

**Figure 1:**
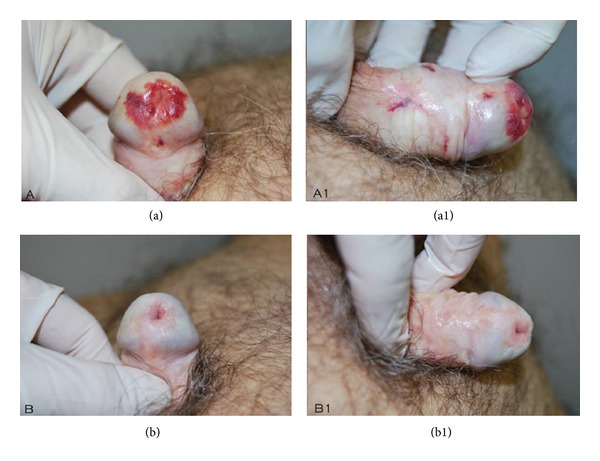
Note (b, b1) the complete remission of the ecchymotic area of the glans and the restitutio ad integrum of foreskin sclerotic ring.

**Figure 2:**
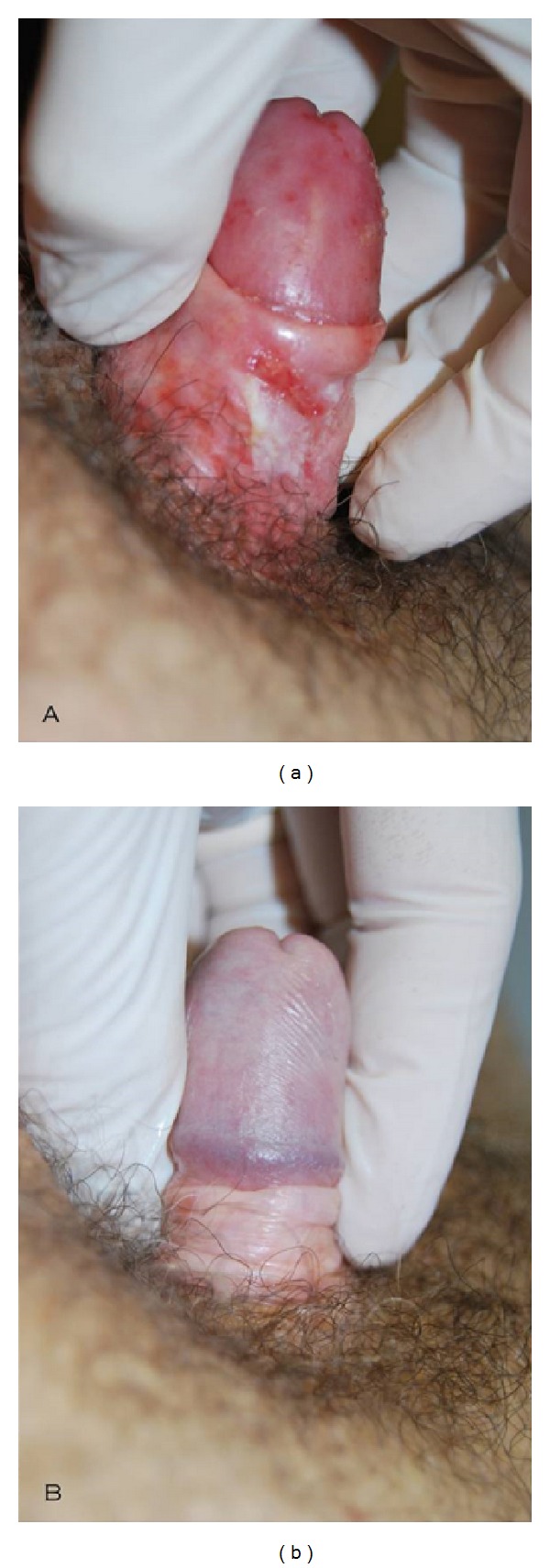
Complete resolution of lichenoid inflammatory area and preputial fissures.

**Figure 3:**
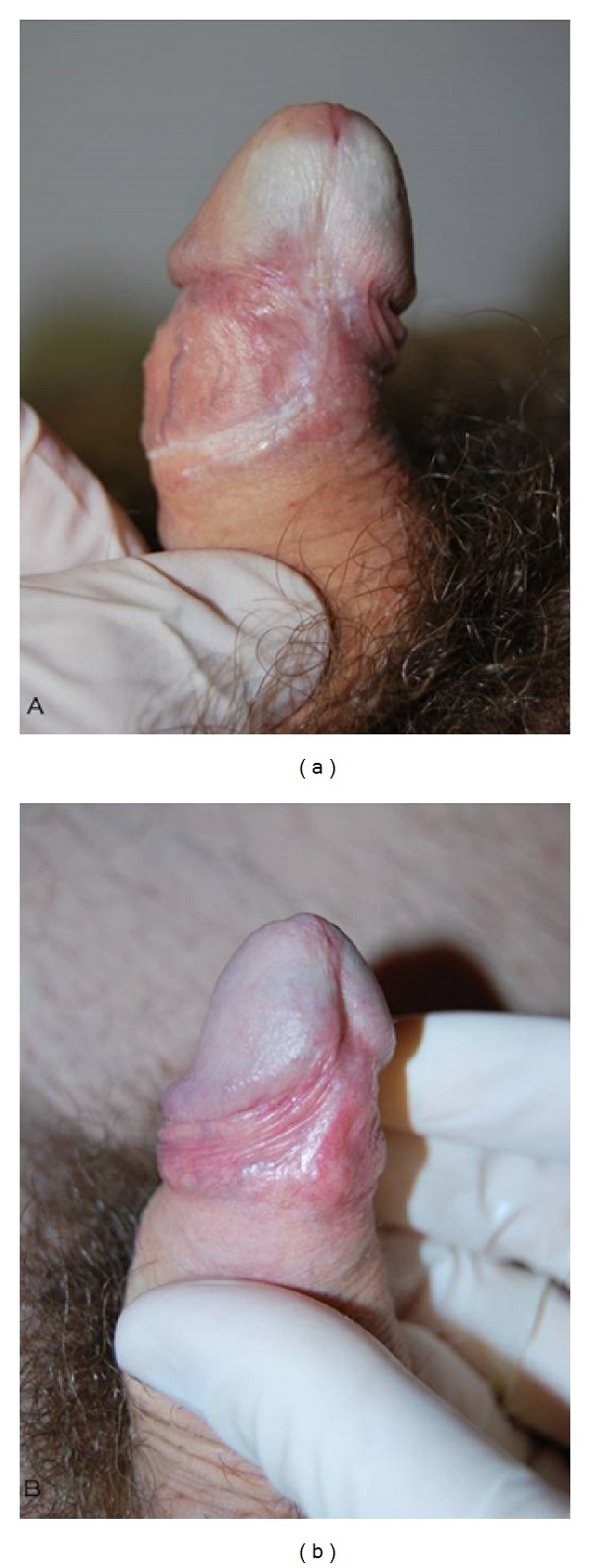
Significant improvement of sclerotic preputial ring and partial repigmentation of the ventral surface of the glans.

After each control, we could see very good improvements in group A patients, while there were moderate improvements in group B patients. The main features highlighted by group A patients were there acquisition of the elastic component of the prepuce and a marked reduction of ecchymoses and infiltrating areas (Figure 1), erythematous areas and fissures (Figure 2), and lichenification areas (Figure 3), in correspondence with the prepuce and glands. After therapy, all group A patients reported to have resumed sexual activity, which was previously impaired by the disease. The therapy sessions were generally well tolerated by all patients. Only a few patients (*n* = 8), during the sessions of infiltration, have felt a sensation of mild pain, especially at the ventral surface of the penis, probably caused by an imperfect action of the anesthetic cream under occlusion.

**Figure 4:**
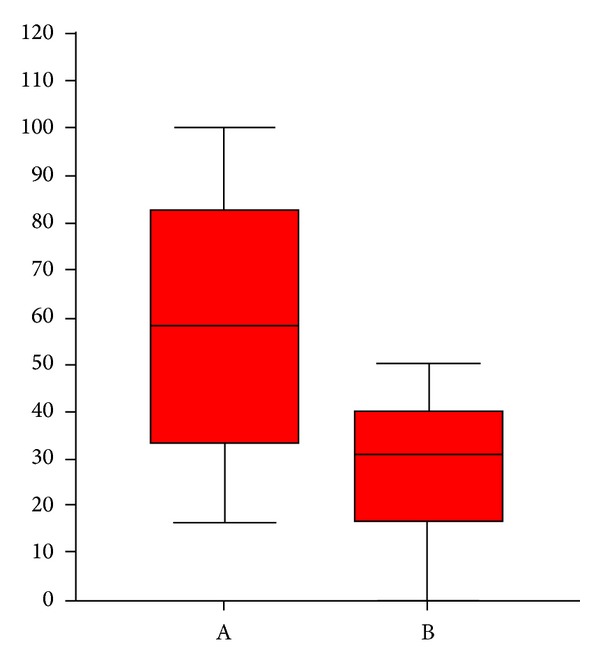
Percentage shown by box plots of changes before and after treatment of the IGA score in the two groups A and B; the differences are evaluated using the Mann-Whitney test (*P* = 0.003).

**Figure 5:**
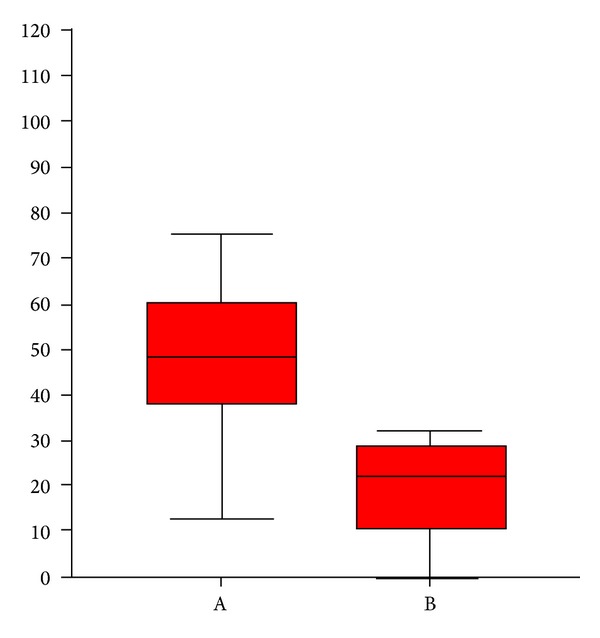
Percentage changes shows by means of box-plot, of the pre and post therapy DLQI score in the two groups A and B, the differences are evaluated using the Mann-Whitney test (*P* < 0.0001).

**Figure 6:**
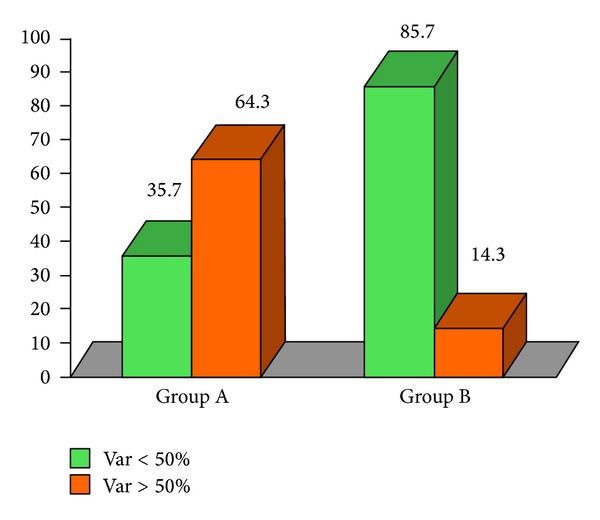
IGA 50% variable: in group A, 9 patients (64.3%) have had a reduction superior or equal to 50% after therapy; in group B, 2 patients (14.3%) have had a reduction superior or equal to 50%.

**Figure 7:**
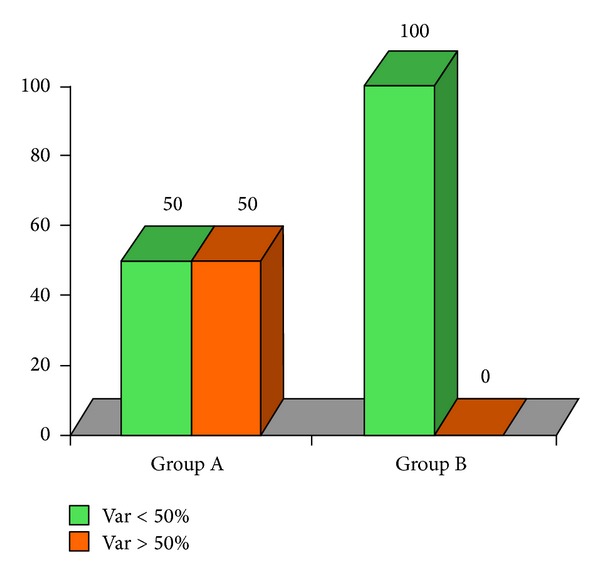
DLQI 50% variable in group A; 7 patients (50%) have had a reduction superior or equal to 50% after therapy with no patients in group B.

At the end of the therapeutic sessions, all group A patients (*n* = 14) experienced a significant improvement of the condition as shown in Table 1. All group B patients showed moderate clinical improvements. Seven patients of group B (*n* = 7/14) declare to have resumed the sexual activity impaired by the disease. After therapy, a statistically significant reduction of the score was found in both groups (Table 1). We could also observe a reduction of IGA (*P* = 0.003, Figure 4) and DLQI score (*P* < 0.0001, Figure 5) in group A greater than in group B. In group A there was a higher percentage of patients (64.3%) with a statistically significant reduction of about more than 50% (*P* = 0.007) of IGA, as compared to group B (14.3%) (Figure 6). In group A, 7 patients had a reduction of DLQI of about more than 50%, while none in group B (Figure 7). There were no other adverse reactions in other group A and group B patients. All patients have shown normal serologic parameters, before, during, and after therapy. The results obtained in group A were maintained until last clinical control (6 months after), while some patients of group B (*n* = 6) have demonstrated slight to moderate representation of pathologic signs, 4–6 months after the end of therapy.

## 4. Discussion

Lichen sclerosus (LS) is a pruritic inflammatory dermatosis, clinically characterized by white atrophic or sclerotic plaques and papules, ecchymoses, erosions, and scarring [12, 13]. The disease has a predilection for genitalia without differences in sex and age. The prevalence of LS is estimated to be up to 1 in 300 with a proportion of affected females to males of about 8 : 1. The exact etiology of the disease is not known but an autoimmune pathogenesis has been supposed. Remarkably, there is a connection with the HLA-subtype, DQ-7, and an increased frequency of other autoimmune disorders including vitiligo, alopecia areata, and thyroid disease. The immunopathology of LS probably involves both T-cell mediated and humoral autoimmune mechanisms. Established areas of lichen sclerosus show a T-lymphocyte dominant inflammatory infiltrate, sometimes with an associated lymphocytic vasculitis. As T-cell receptor gene rearrangement studies from affected vulvar and penile skin were performed, a monoclonal group of rearranged g-chain gene of the T-cell receptor was recognized, which could explain the increased risk of malignant change in patients suffering from LS [14]. The clinical characteristic appearance of male LS consists of ivory-white plaques and atrophic or thickened skin, usually associated with excoriations and ecchymoses due to both repeated scratching and the reduction of elasticity, and often associated with a phimosis. Itch could be manifested in the late phase of pathology and both scratching and inflammation could lead to hyperkeratosis and superficial erosions. Because of these characteristics, we hypothesized that PDRN could play a role in reducing the primary cause of lichen sclerosus, the inflammation of the tissue, and in part, inducing tissue regeneration. We therefore wanted to challenge some of the regenerative effects demonstrated by PDRN in association with anti-inflammatory effects demonstrated by CP 0.05% cream that seemed to be useful to counteract the progression of LS. In particular, preliminary experiments showed that PDRN determined a statistically significant increase in fibroblast growth, as reported by Sini et al. [1]. Fibroblasts are important precursors of collagens, glycosaminoglycans, and elastic and reticular fibers glycoproteins that can be found in extracellular matrix. Stimulation of these cells may play a key role in tissue regeneration in the course of LS. Again, it has been proved that PDRN plays a role in the reduction of proinflammatory cytokines, which leads to an anti-inflammatory effect in several autoimmune diseases, as reported by Bitto et al. [14]. It is already known that the primary pathogenetic path of LS is the skin inflammation. The demonstrated [15] anti-inflammatory effect of PDRN, bound to the reduction of cytokine production [14], may impact in some way the progression of the disease, counteracting the *primum movens *due to several pathological effects. Last, but not least, we have demonstrated the effects of PDRN in promoting growth and differentiation of human preadipocytes [2], which constitute the largest and most accessible reserves of adult stem cells. These cells are known to have an effect on the regeneration and tissue repair and on cellular aging; this could be another positive action performed by PDRN in the pathogenesis of LS. CP 0.05% cream, a potent topical corticosteroid, which is considered to represent a first-line therapy for lichen sclerosus [10] with a marked anti-inflammatory action and with a favorable benefit/risk ratio, has emerged as topical treatment of various inflammatory and immunological skin disorders, such as psoriasis, lichen ruber planus, and lichen sclerosus [15–19]. CP shows marked effects in the reduction of immunological skin inflammatory infiltrate. The association between PDRN and CP may allow the use of a lower dose of topical corticosteroids in patients suffering from LS, with a greater compliance as compared to only ultrapotent topical corticosteroid, usually used for long-term therapy in course of lichen sclerosus. Our clinical experience, has just recently shown [20] that the polydeoxyribonucleotides, in combination with topical corticosteroids, may be useful in the medium term, in patients suffering from lichen sclerosus. Our current study, in addition to our previous one [21], has demonstrated the statistic differences in effectiveness of adjuvant polydeoxyribonucleotides in combination with first-line topical corticosteroids therapy, compared to patients who used only the first-line topical corticosteroid.

This result may be caused by a double action, which could impact two basic pathways that characterize the pathogenesis of lichen sclerosus: the chronic inflammatory path and the alteration of cell differentiation, in course of lichen sclerosus. In other words, the synergy between both anti-inflammatory effects related to the CP (ultrapotent steroid) and the demonstrated regenerative action of PDRN [1–9] can lead to a better outcome in clinical terms. This observation, borne out in our clinical experience, according to other authors [21], confirms that a multitherapeutic and more aggressive approach, in the course of early and acute lichen sclerosus, could make the best results both in clinical terms and in increasing of the remission time of pathological signs.

Although this therapy has proven to be effective, we believe it is appropriate to limit the application in a few sessions. our further experience, in combination with further scientific studies, will tell us if in the future it will be possible to think of a pulsed therapy for the maintenance of clinical results.

## 5. Conclusions

Despite many therapeutic approaches proposed over the years, the definitive treatment of lichen sclerosus is still being codified [10]. We believe that a viable therapy consists of a multidisciplinary approach that is based on proper clinical staging and histological diagnosis. We maintain that a valid therapeutic proposal is not confined to a drug, but to a therapeutic set and to a proper therapeutic approach, based on the type and the severity degree of the disease. In this context, through this preliminary study, and for the first time in this dermatological disease, we have highlighted the efficacy, tolerability, and safety profile demonstrated by PDRN in association with CP, which could be cited, (if further studies confirm these early data) as one of the effective adjuvant therapies in the management of lichen sclerosus.

## 6. Summary

Lichen sclerosus (LS) is a complex chronic inflammatory skin disease, with a predilection for genital skin. The exact pathogenesis of LS remains unclear, but it is generally regarded as an autoimmune pathogenetic pathway. The histopathology of LS suggests abnormalities in extracellular matrix composition and a huge immunological skin inflammation, which leads to sclerosis and tissue atrophy. In this study we have evaluated the clinical results of local subdermal administration of polydeoxyribonucleotide (PDRN) in LS lesions. PDRN, an A2A adenosine receptor agonist, has shown anti-inflammatory efficacy, due to the reduction of cytokine production and the regenerative effects associated with the growth of human preadipocytes. PDRN also enhanced the expression of vessel endothelial growth factor and the stimulation of fibroblast. Clobetasol propionate (CP) a synthetic corticosteroid with a marked anti-inflammatory action has emerged as a topical medication for treatment of inflammatory and immunological skin disorders. PDRN + CP therapy shows a higher anti-inflammatory and skin regeneration effect, associated with good compliance among patients and a dramatic clinical improvement, according to visual IGA and DLQI assessment.

## Figures and Tables

**Table 1:** Values median (range) of IGA and DLQI scores before and after therapy in group A and group B; comparisons are evaluated using paired Wilcoxon signed-rank for paired data.

Variables	Group A			Group B		
	Before therapy	After therapy	*P* value	Before therapy	After therapy	*P* value
IGA Median (range)	6 (3–10)	2 (0–7)	0.001	5 (3–9)	4 (2–6)	0.001
DLQI Median (range)	15 (8–19)	7 (3–11)	0.001	14 (8–19)	11 (6–16)	0.003
